# Comparison of the efficacy and safety of removing bandage contact lenses on the fourth and seventh postoperative day after transepithelial photorefractive keratectomy

**DOI:** 10.1016/j.heliyon.2023.e21129

**Published:** 2023-10-17

**Authors:** Hangshuai Zhou, Yanhua Jin, Gengmin Tong, Guangjin Zhao, Hongyan Wu

**Affiliations:** Department of Ophthalmology, Dongyang People's Hospital, Dongyang, China

**Keywords:** Bandage contact lenses, Transepithelial photorefractive keratectomy, Myopia, Eye discomfort

## Abstract

**Purpose:**

To compare the differences in the removal of bandage contact lenses (BCLs) at 4 and 7 days after transepithelial photorefractive keratectomy (TransPRK) in term of visual rehabilitation, eye discomfort, and postoperative complications.

**Methods:**

This retrospective cohort study included patients with myopia undergoing TransPRK; in Group 1, the BCLs were removed on the 4th postoperative day, while in Group 2, the BCLs were removed on the 7th postoperative day. All patients underwent a 6-month follow-up, including slit-lamp examination and visual acuity assessment. Subjective evaluations of pain and eye discomfort were recorded after the BCLs removal.

**Results:**

In total, 376 eyes of 191 patients in Group 1 and 346 eyes of 177 patients in Group 2 were enrolled. The two groups were matched for sex, age, preoperative corrected distance visual acuity, and tear film break-up time. Patients in Group 1 exhibited slightly lower levels of myopia, resulting in a shallower ablation depth and shorter ablation time than those in Group 2. No statistically significant differences in visual acuity recovery, haze severity, and incidence of infectious keratitis were observed within 6 months after surgery between the two groups. However, patients in Group 2 experienced significantly fewer discomfort symptoms (discharge, foreign body sensation, and blurred vision) after BCLs removal than patients in Group 1 and had fewer postoperative complications (recurrent corneal epithelial erosion).

**Conclusion:**

Delayed removal of the BCLs one week after TransPRK effectively alleviated early discomfort symptoms and reduced the risk of recurrent corneal epithelial erosion without increasing the likelihood of infectious keratitis.

## 1 Introduction

Refractive error is a common cause of visual impairment worldwide, and corneal refractive surgery is one of the most frequently used surgical approaches for correcting refractive errors [1,2]. Surface ablation has become increasing popular in recent years owing to its improved safety, especially in patients with thin corneas and high myopia [3]. Multiple procedures can be categorized as surface ablations, including photorefractive keratectomy (PRK), laser-assisted subepithelial keratomileusis (LASEK), and off-flap epiploic-laser *in situ* keratomileusis (Epi-LASIK). However, surface ablation is not accepted by a subset of patients owing to slower visual recovery and increased postoperative discomfort [4,5]. Recently, single-step transepithelial photorefractive keratectomy (TransPRK; SCHWIND eye-tech-solutions GmbH, Kleinostheim, Germany), which ablates the epithelium and stroma in a single step, has become a popular treatment option in the corneal refractive surgery field [6,7]. Previous studies have reported that this treatment profile can reduce the haze formation, epithelial healing period, and early postoperative pain compared with conventional PRK and LASEK [[8], [9], [10]].

Bandage contact lenses (BCLs) have been shown to relieve pain, protect de-epithelialized corneas, and accelerate corneal re-epithelialization. Currently, BCLs are the standard treatment for pain control during epithelial healing during the first few days after TransPRK [11,12]. The corneal epithelial healing process usually takes four days [13,14], and the BCL is removed after the epithelial defect has healed. Delayed BCL removal is hypothesized to result in a higher risk of infection; however, BCL can be maintained longer with the prophylactic use of topical antibiotics, which may provide more stable and enhanced epithelial healing and reduce discomfort.

This study aimed to evaluate the differences in visual rehabilitation, ocular discomfort, and postoperative complications with BCLs removal 4 and 7 days after TransPRK.

## 2 Methods

This retrospective comparative study included patients with myopia who underwent TransPRK surgery with BCLs removed on the fourth and seventh postoperative day in Group 1 and 2, respectively. Patients were recruited between January 2022 and December 2022 at Dongyang People's Hospital, China. Inclusion criteria for both groups were age >18 years, myopic spherical equivalent (SE) refraction (range: −0.75 D to −10.00 D), and stable myopia for more than one year. The exclusion criteria were astigmatism >4 D; keratometry >48 D; corneal thickness <480 μm; mesopic pupil size >7 mm; abnormal or keratoconus topography; and patients with inflammatory or infectious corneal disease, pregnancy, diabetes mellitus, connective tissue disease, and any other systemic diseases that could affect corneal wound healing. All the patients were asked to stop wearing hard contact lenses for 4 weeks or soft contact lenses for 1 week before surgery. Before recruitment, all eligible patients were consulted and informed about the benefits and risks of the technique. The study adhered to the tenets of the Declaration of Helsinki and ethical approval was provided by the Institutional Ethics Committee of Dongyang People's Hospital.

### 2.1 Preoperative and postoperative assessment

Preoperative assessments included autorefraction, cycloplegic subjective refraction, manifest refraction, uncorrected distance visual acuity (UDVA), corrected distance visual acuity (CDVA), slit-lamp examination, keratometry, Scheimpflug-based corneal topography (Pentacam HR; Oculus Optikgeräte, Wetzlar, Germany), ophthalmoscopy, intraocular pressure measurement, mesopic pupil diameter, and tear film break-up time. Corneal wavefront aberrations were measured using Keratron Scout (Optikon 2000, Rome, Italy).

The patients attended follow-ups at 1 day, 2 days, 5 days, 1 week, 1 month, 3 months, and 6 months after surgery. A slit-lamp examination was performed at each follow-up visit to explore any signs of epithelial defects, corneal clarity, filiform keratitis, filamentary keratitis, or other complications. After healing of the corneal epithelial defect, the BCLs were removed on postoperative days 4 or 7. The patients also completed questionnaires regarding ocular discomfort and pain after BCLs removal. A visual analog scale (VAS) was used to determine the degree of pain, with 0 indicating no pain and 10 indicating the worst pain experienced by the patient. Eye discomfort (including discharge, epiphora, foreign body sensation, and blurred vision) was assessed on a scale of 0–10, where 0 indicated no complaint at all and 10 indicated the worst possible complaint. At subsequent visits, the UDVA was assessed using the Snellen chart and converted to the Logarithm of the Minimum Angle of Resolution (LogMAR) scale at 14 days, 1 month, 3 months, and 6 months after surgery. Early postoperative complications, including infectious keratitis, recurrent corneal epithelial erosion, and filamentary keratitis within six months after surgery, were recorded in both groups. Postoperative corneal haze at 1, 3, and 6 months was graded by two independent ophthalmologists under a slit-lamp microscope according to the Fantes scale [15], and the average of the two values was calculated.

### 2.2 Surgical procedure

All the eyes underwent single-step TransPRK performed by the same surgeon (ZHS) using a SCHWIND Amaris 500 S excimer laser platform (SCHWIND eye-tech-solutions GmbH, Kleinostheim, Germany). Preoperative anesthesia was achieved with proparacaine drops (0.5 %, Alcaine®) instilled 2 times, commencing 10 min before surgery. Following disinfection of the eyelid skin, the eye area was scrubbed and draped, a lid speculum was inserted between the eyelids of the eye to be treated, and the other eye was blocked. Epithelial and stromal ablations were performed using a single continuous profile on an excimer laser platform. Based on previous epithelial characterization studies, epithelial ablation was targeted 55-mm and 65-mm, centrally and peripherally, respectively [16]. Postoperatively, Mitomycin C 0.02 % was applied to the stromal surface for 20 s, followed by washing with 20 ml cold saline. After instillation of one drop of 0.5 % topical levofloxacin at the surgical site, a BCL (Acuvue Oasys; Johnson & Johnson, Jacksonville, FL, USA) was placed on the cornea for 4 or 7 days. After surgery, all the eyes received 0.5 % topical levofloxacin four times a day until removal of the contact lens, fluorometholone (0.1 %) drops four times a day for 4 months, and artificial tears drops four times a day for 4 months.

### 2.3 Analysis

All the statistical tests were performed using IBM SPSS Statistics for Windows version 23. The Student's t-test was used to compare descriptive data between the two groups. Group differences in continuous data were examined using the Mann-Whitney *U* test or independent samples *t*-test. If both eyes underwent surgery, the data from both eyes were included in the study. All the data are presented as the mean ± standard deviation. A *p*-value <0.05 was considered statistically significant.

## 3 Results

The study included 722 eyes of 368 patients: 376 eyes of 191 patients in Group 1 and 346 eyes of 177 patients in Group 2. The two groups were matched for sex, age, preoperative CDVA, and tear film break-up time. However, patients in Group 1 exhibited slightly lower levels of myopia, resulting in a shallower ablation depth and shorter ablation time than those in Group 2 (Table 1). All the patients completed a 6-month follow-up examination.

**Table 1:** Baseline preoperative values.

Variable	Group 1	Group 2	P value
Number of eyes	376	346	NA
Sex (n), male/female	98/93	88/89	0.760
Age(years)(Mean ± SD)	24.93 ± 6.34	25.17 ± 6.85	0.622
Preoperative SE（D）(Mean ± SD)	−4.63 ± 1.48	−4.95 ± 1.70	0.013
Preoperative CDVA （Log MAR）(Mean ± SD)	−0.04 ± 0.07	−0.03 ± 0.08	0.707
Ablation depth（um）(Mean ± SD)	131.87 ± 18.86	135.64 ± 20.64	0.014
Ablation time (S) (Mean ± SD) Tear film break-up time(S) (Mean ± SD)	59.39 ± 7.63 8.26 ± 3.03	60.37 ± 9.96 7.94 ± 2.91	0.019 0.144

SD: standard deviation, CDVA: corrected distance visual acuity.

### 3.1 Visual acuity

On the 14th day after the operation, 25.5 % of the eyes in Group 1 had achieved 0.0 LogMAR UDVA or better, compared to 21.4 % of the eyes in Group 2, who had reached the same level of visual acuity. However, the difference between the two groups was not statistically significant (*p* = 0.19). Six months after the surgery, the percentage of eyes with 0.0 LogMAR UDVA or better had significantly increased in both groups. In Group 1, 96.8 % of the eyes achieved this level of visual acuity, whereas in Group 2, 98.6 % of the eyes achieved the same level. However, the difference between the two groups was not statistically significant (*p* = 0.12).

**Fig. 1:**
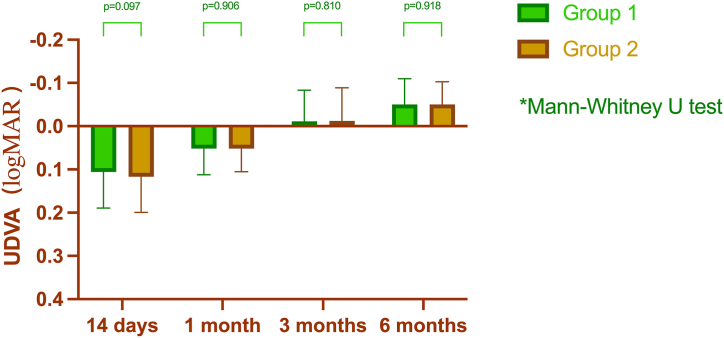
The difference in uncorrected visual acuity between groups within 6 months after surgery.

In Group 1, patients had an UDVA of 0.11 ± 0.08, 0.05 ± 0.06, −0.01 ± 0.07, and −0.05 ± 0.06 at 14 days, 1 month, 3 months, and 6 months postoperatively, respectively. Meanwhile, in Group 2, the UDVA was 0.12 ± 0.08, 0.05 ± 0.05, −0.01 ± 0.08, and −0.05 ± 0.05 at 14 days, 1 month, 3 months, and 6 months after surgery, respectively. No significant difference in the uncorrected visual acuity was observed between the two groups at the four designated time points (Fig. 1).

### 3.2 Pain and eye discomfortable scores

**Fig. 2:**
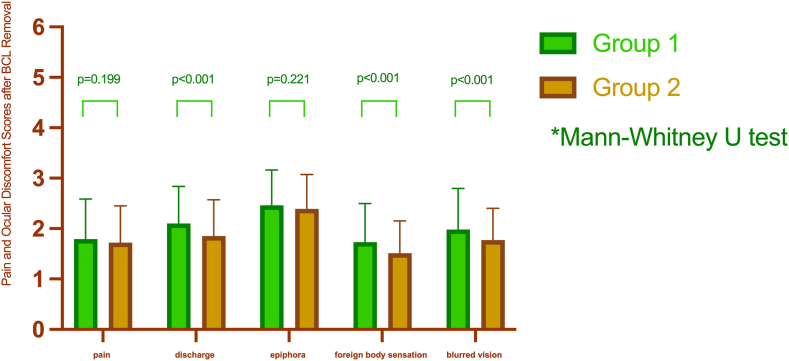
The difference in pain and eye discomfortable scores between groups after BCLs removal.

After the BCLs were removed, individuals in Group 1 reported higher scores for discharge, foreign body sensation, and blurred vision (2.10 ± 0.74, 1.73 ± 0.77, and 1.98 ± 0.82, respectively) compared to those in Group 2 (1.85 ± 0.72, 1.51 ± 0.64, and 1.77 ± 0.63, respectively); these differences were statistically significant (Fig. 2). However, no significant difference was observed in the pain and epiphora scores between the two groups after the BCLs were removed. Group 1 had pain and epiphora scores of 1.79 ± 0.80 and 2.46 ± 0.70, respectively, while Group 2 had scores of 1.72 ± 0.73 and 2.39 ± 0.68, respectively.

### 3.3 Haze

**Fig. 3:**
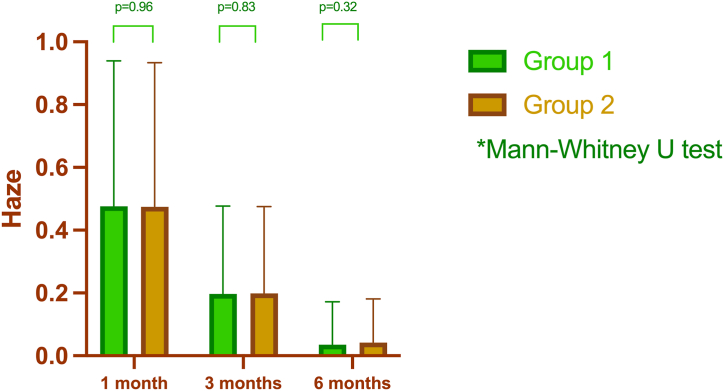
The difference in the haze between groups within 6 months after surgery.

Haze scores were assessed at 1 month, 3 months, and 6 months postoperatively in both the groups. However, the statistical analysis did not indicate any significant differences between the two groups at any time points. The haze scores were 0.47 ± 0.46, 0.20 ± 0.28, and 0.04 ± 0.14 for Group 1, and 0.48 ± 0.46, 0.20 ± 0.28, and 0.04 ± 0.14 for Group 2, respectively (Fig. 3). No discernible correlation was observed between the postoperative haze scores and either the ablation time or depth at any point during the study.

### 3.4 Postoperative complications

Within 6 months after surgery, Group 1 had one case of infectious keratitis, six cases of filamentary keratitis, and 25 cases of recurrent corneal epithelial erosion, whereas Group 2 had one case of infectious keratitis, three cases of filamentary keratitis, and five cases of recurrent corneal epithelial erosion. No statistically significant difference was observed in the incidence of infectious and filamentary keratitis between the two groups, however, a statistically significant difference was observed in the incidence of recurrent corneal epithelial erosion (Table 2).

**Table 2:** Post-operative complications.

Parameter	Group 1(N%)	Group 2(N%)	P value
Infectious keratitis	1(0.3)	1(0.3)	0.953
Recurrent corneal epithelial erosion	25(6.6)	5(1.4)	<0.001
Filamentary keratitis	6(1.6)	3(0.9)	0.378
Total	36(9.6)	13(3.8)	0.002

N = number of cases.

## 4 Discussion

The TransPRK technique was first introduced by Alio [17] in the early 1990s; however, its popularity has increased significantly after studies conducted by Aron-Rosa et al. [18] and Gimbel et al. [19], in which both the corneal epithelium and stroma were ablated using an excimer laser instead of traditional mechanical or chemical debridement techniques. This approach is often preferred by both patients and physicians owing to the provision of precise and controlled ablation, while reducing the risk of complications associated with mechanical or chemical debridement. Subsequently, due to the excision of the corneal epithelium and partial corneal stroma during the surgery, patients in the early stages after PRK may experience symptoms similar to photokeratitis, such as photophobia and tearing [20]. In 2017, the Amaris single-step platform was enhanced with a new software feature called “smart-pulse technology,” aimed at improving the outcomes of refractive surgeries [21]. This laser pulse technology software algorithm was designed to reduce surface irregularities of the corneal stromal bed immediately after surgery. The laser spot distribution uses a truncated super-Gaussian spot shape, and the platform avoids the thermal load and ablation impact of lateral pulse technology. Additionally, it utilizes a 3-dimensional geometric model that more accurately represents the curvature of the cornea, resulting in a smoother corneal stromal bed, which is expected to facilitate better recovery and faster re-epithelialization [22].

The postoperative use of a protective bandage lens can help safeguard the cornea following ocular surface surgery [23,24]. Bandaged soft contact lenses are commonly used in surface ablation surgery to promote epithelial healing, reduce pain, and prevent corneal haze formation [25,][26]]. However, the optimal timing for removing the bandage lens remains unclear. It is typically removed once the corneal epithelium has healed. Recent studies have suggested that delaying the removal of the bandage lens may be beneficial in patients undergoing alcohol-assisted PRK. Specifically, two studies found that removing the bandage lens either 5 days or 7 days after surgery could reduce postoperative complications compared to removing it 3 or 4 days after surgery [26,27]. In both the studies, the corneal epithelium was removed using alcohol-assisted methods. Based on the belief that the corneal epithelial healing time after TransPRK is significantly shorter than that after alcohol-assisted PRK [28], they conducted a retrospective study to compare the efficacy and safety of bandage lens removal at 4 and 7 days following TransPRK. No significant differences were observed in the visual acuity and haze between the two groups. However, removal of the BCLs seven days after the surgery resulted in a notable reduction in postoperative discomfort symptoms and recurrent corneal epithelial erosion. Although patients in Group 2 had higher degrees of myopia, this was presumed to require more stromal ablation and postoperative discomfort [29,30]. One significant reason for wearing BCLs for extended periods is that they promote healing of the epithelium, improve anchorage to the underlying layers, and create smoother epithelial surfaces [31]. BCLs provided a protective environment for the fragile, newly formed epithelium, which aids in the healing process. Additionally, BCLs can help reduce discomfort by minimizing friction between the eyelid and the cornea. These benefits ultimately result in tighter and more complete corneal healing.

In this study, one case of infectious keratitis occurred in both the groups. Previous research has suggested that wearing BCLs after corneal refractive surgery might increase the risk of infectious keratitis, which might further increase with a longer duration of BCLs use [32]. However, this study found that prolonged use of BCLs did not increase the risk of infectious keratitis, as the corneal epithelium healed in most patients within 4 days after surgery, and patients were administered antibiotic eye drops before removing the BCLs. Additionally, infectious keratitis following TransPRK is usually associated with a favorable prognosis [33], and both cases of infectious keratitis in this study were successfully treated, and resulting in a final UDVA of at least 0.0 LogMAR.

In addition to the duration of wear, the material used for the BCLs is a crucial factor. Senofilcon A was selected because owing to its ability to reduce postoperative pain and discomfort more effectively than other materials [34,35]. Taylor et al. [36] used high-magnification microscopy to evaluate the geometry of different types of contact lenses and found that the Senofilcon A lens had a tapered edge with the thinnest profile among the studied lenses. The authors postulated that this unique geometry could explain the improved pain control observed using this type of lens. Furthermore, a study by AlDahash et al. [12] demonstrated that exchanging the bandage lens one day after TransPRK surgery resulted in a reduction in postoperative pain compared to leaving the same lens in place. The authors hypothesized that inflammatory mediators might adhere to the bandage lens, and that removing the lens could potentially reduce inflammation and pain.

The limitations of this study include a short follow-up of only six months, non-randomized retrospective design, and lack of functional visual quality evaluation and postoperative residual refraction differences. However, this study has many clinical implications, as it is the first to compare the differences in the efficacy and safety of removing BCLs at different time points after TransPRK. Compared with removing the bandage lens 4 days after surgery, removing the bandage lens 7 days after surgery was effective in reducing recurrent corneal epithelial erosion and discomfort after removing the bandage lens. Despite the differences in preoperative refraction, ablation time, and depth, the study further highlights the advantages of removing the BCLs 7 days after surgery.

In summary, although the corneal epithelial healing time following TransPRK surgery was faster than that following conventional PRK surgery, retaining the BCLs for one week effectively alleviated early discomfort symptoms after removal of the BCLs and decreased the likelihood of recurrent corneal epithelial erosion without increasing the risk of infectious keratitis.

## Ethics declarations

This study was reviewed and approved by the ethical committee of the Dongyang People's Hospital, with the approval number: dongrenyi 2013-YX-043. Informed consent was not required for this study because it was a retrospective study.

## Data availability statement

Due to privacy protection reasons, we have temporarily not shared the data in a publicly available repository. Data will be made available on request.

## CRediT authorship contribution statement

**Hangshuai Zhou:** Conceptualization, Project administration, Writing – original draft, Writing – review & editing. **Yanhua Jin:** Investigation, Methodology. **Gengmin Tong:** Data curation, Writing – review & editing. **Guangjin Zhao:** Investigation, Project administration, Resources. **Hongyan Wu:** Data curation, Investigation, Resources.

## Declaration of competing interest

The authors declare that they have no known competing financial interests or personal relationships that could have appeared to influence the work reported in this paper.
