# Supplementary material for: Comparison of the efficacy and safety of removing bandage contact lenses on the fourth and seventh postoperative day after transepithelial photorefractive keratectomy
Source: Heliyon. 2023 Oct 17;9(10):e21129. doi: 10.1016/j.heliyon.2023.e21129 (PMC10597848; doi:10.1016/j.heliyon.2023.e21129)
Supplement: Multimedia component 1 [file mmc1.docx]

Thank you for taking the time to complete this survey. We are interested in gathering information about your experience with pain, discharge, epiphora, foreign body sensation, and blurred vision. Please rate each item on a scale from 0 to 10, where 0 means you do not experience this symptom, and 10 means severe and greatly affects your daily life.

1. How would you rate the level of pain you are experiencing? ( )

0 1 2 3 4 5 6 7 8 9 10

1. How much discharge are you currently experiencing, such as mucus or secretions? ( )

0 1 2 3 4 5 6 7 8 9 10

1. How would you rate the amount of epiphora you are experiencing? ( )

0 1 2 3 4 5 6 7 8 9 10

1. Are you experiencing a foreign body sensation in your eye, such as feeling like there is something in it? If so, how would you rate the level of discomfort? ( )

0 1 2 3 4 5 6 7 8 9 10

1. Are you experiencing blurred vision, such as difficulty seeing objects clearly or focusing your eyes? If so, how would you rate the level of blurriness? ( )

0 1 2 3 4 5 6 7 8 9 10

Thank you for taking the time to complete this survey. Your feedback is greatly appreciated.
